# Antibody Response to the Furin Cleavable Twenty-Seven Amino Acid Peptide (p27) of the Fusion Protein in Respiratory Syncytial Virus (RSV) Infected Adult Hematopoietic Cell Transplant (HCT) Recipients

**DOI:** 10.3390/vaccines8020192

**Published:** 2020-04-21

**Authors:** Xunyan Ye, Wanderson Cabral de Rezende, Obinna Patrick Iwuchukwu, Vasanthi Avadhanula, Laura L. Ferlic-Stark, Kirtida D. Patel, Felipe-Andres Piedra, Dimpy P. Shah, Roy F. Chemaly, Pedro A. Piedra

**Affiliations:** 1 Department of Molecular Virology & Microbiology, Baylor College of Medicine, Houston, TX 77030, USA; xunyan.ye@bcm.edu (X.Y.); Obinna.Iwuchukwu@bcm.edu (O.P.I.); avadhanu@bcm.edu (V.A.); lferlic@bcm.edu (L.L.F.-S.); kpatel@bcm.edu (K.D.P.); Felipe-Andres.Piedra@bcm.edu (F.-A.P.); 2 Department of Pharmacology, Baylor College of Medicine, Houston, TX 77030, USA; rezende@bcm.edu; 3 Department of Epidemiology & Biostatistics, The University of Texas Health Science Center at San Antonio, San Antonio, TX 78229, USA; ShahDP@uthscsa.edu; 4 Departments of Infectious Diseases, Infection Control & Employee Health, The University of Texas MD Anderson Cancer Center, Houston, TX 77030, USA; rfchemaly@mdanderson.org; 5 Department of Pediatrics, Baylor College of Medicine, Houston, TX 770030, USA

**Keywords:** respiratory syncytial virus, p27 antibody, hematopoietic cell transplant recipients

## Abstract

*Background:* Cleavage of the inactive precursor fusion protein (F0) of respiratory syncytial virus (RSV) at two furin-recognition sites is required for membrane fusion activity, and the cleavage releases the twenty-seven amino acid peptide (p27). However, a recent study shows that p27 was an immunodominant epitope in RSV infected children, indicating that p27 was recognized as an immunogen. In the present study, we investigated the immunogenicity of p27 in an immunocompromised population of adults by measuring serum and mucosal antibody responses to p27 in samples from adult hematopoietic cell transplant (HCT) recipients. *Methods:* We prospectively enrolled a cohort of RSV infected HCT recipients. Serum and nasal-wash samples were obtained within the first week of RSV infection (acute) and 3 to 5 weeks post-infection (convalescent). We quantified the serum and mucosal IgG and IgA anti-p27 antibodies by a RSV/A p27 peptide enzyme-linked immunosorbent assay (ELISA) and serum and mucosal p27 like antibodies (P_27_LA) by a p27 competitive antibody (P_27_CA) assay. *Results:* The lower limit of detection for the ELISA and P_27_CA assays was 0.2 and 50 ng/mL, respectively with no cross-reaction detected with a panel of monoclonal antibodies targeting pre-fusion and post-fusion antigenic sites. P27 antibodies were detected at nanogram concentration in sera and nasal washes in the majority of RSV infected HCT recipients. However, there was no significant difference in the geometric mean antibody concentrations between the acute and convalescent sera (except for serum P_27_LA), between HCT recipients who shed RSV <14 days and ≥14 days, as well as between RSV/A and RSV/B infected HCT recipients. In addition, approximately 30% of HCT recipients had a 4-fold or greater decrease in mucosal IgG and IgA anti-p27 antibodies during viral clearance. *Conclusion:* In conclusion, in RSV naturally infected adult HCT recipients, the antibodies against p27 were detectable in both serum and nasal wash samples with higher concentration in serum than that in nasal washes. However, nearly 30% of RSV infected HCT recipients had a significant decrease in their mucosal anti-p27 antibody, suggesting that IgG and IgA anti-p27 antibodies were binding to either free viruses or RSV infected cells containing p27, and that anti-p27 antibodies in the respiratory tract were part of the mucosal antibody response in controlling RSV infection.

## 1. Introduction

Respiratory syncytial virus (RSV) is a common respiratory virus that can infect people of all ages. The outcomes of RSV infections depend on the patient population. RSV infection accounts for substantial morbidity and mortality among infants and older adults [1,2,3,4], although the infection rate is much lower among older adults compared to infants. A population vulnerable to severe RSV infection is hematopoietic cell transplant (HCT) recipients [5,6]. In contrast to immunocompetent adults, RSV infected HCT recipients are much more likely to present prolonged viral shedding and duration of illness [7]. Host and transplant related factors in HCT recipients, such as smoking history, the type of conditioning regimen, and absolute lymphocyte or neutrophil count appear to be major risk factors for disease progression to pneumonia more than viral factors, such as post-transplant recipient RSV neutralizing antibody levels and infecting RSV subtypes [8]. 

There is a major medical need for an effective intervention against RSV. Currently, there is no approved vaccine for RSV despite over 60 years of research. Inhaled ribavirin, a guanosine analog, is the only FDA-approved drug for treatment of hospitalized infants and young children with RSV bronchiolitis [9]. But because of its cost and controversial benefit, ribavirin is rarely used [10]. Palivizumab (Synagis; MedImmune), a recombinant humanized monoclonal antibody, is the only FDA-approved immunoprophylaxis for RSV infection in a select group of premature high-risk infants and those with chronic cardiopulmonary disease [11,12,13,14]. Palivizumab does not work as therapeutic drug once the RSV infection is established. There is a critical need to develop a well-tolerated and effective vaccine and antiviral drug to prevent disease caused by RSV infections. 

Most vaccine candidates and antiviral drugs in development target the fusion (F) protein [15]. The F protein is one of two major surface glycoproteins of RSV virions. It is initially synthesized as a 70 kDa inactive precursor (F0), which possesses two furin cleavage sites (site I, RARR109, and site II, KKRKRR136). F0 undergoes cleavage by furin-like enzymes during intracellular maturation in the trans Golgi apparatus. It results in disulfide-linked F1 (50 kDa) and F2 (20 kDa) subunits releasing 27 amino acids (109–136) peptide (p27) [16,17]. Therefore, the mature pre-fusion F protein on infected cells or on the surface of the virions is assumed not to contain p27. The location of p27 on the F0 and the length of it, but not the sequence of it, are highly conserved in all human RSV strains. Proteolytic processing at both cleavage sites of the F protein is required by RSV to induce syncytium in transfected cells [16]. The fully activated F protein is in a prefusion conformation containing potent neutralization epitopes on the F1 and F2 subunits. Current vaccine development efforts are often focused on generating a prefusion F protein [18,19,20], and few efforts are focused on the F protein containing the p27 epitope as a partially cleaved F (prefusogenic F). A recent study showed that among the 14 linear and conformational epitopes on the RSV F protein expressed from whole genome-fragment phage display libraries, the p27 peptide (aa 101–121) demonstrated the strongest binding to a panel of sera from children <2 years old [21], indicating p27 is an immunodominant epitope in RSV infected children. A recent study from our group showed that antibody binding to peptide (aa 101–157) containing p27 was statistically higher in the nasal washes of early-recovered HCT recipients compared with late-recovered HCT recipients [22], indicating again p27 is a immunodominant epitope in RSV infected HCT adults. In the present study, we investigated the serum and mucosal antibody response to p27 in RSV infected HCT adult recipients. 

## 2. Materials and Methods

### 2.1. Study Subjects

We have previously described the RSV-F antigenic site-specific serum antibody responses in RSV infected HCT recipients [23,24]. In this report, we extend our findings to include the serum and mucosal antibody response to p27 from RSV/A. In brief, the 33 HCT recipients with laboratory-confirmed RSV upper respiratory tract infections at enrollment and negative chest radiographic findings were enrolled within 72 hours of RSV diagnosis between January 2012 and April 2015 [23,24]. The RSV infected HCT recipients were stratified by level of risk for progression to the lower respiratory tract as previously described [23,24]. To evaluate serum and mucosal antibodies against p27, serum and nasal wash samples were collected at enrollment (acute samples) and 14-60 days after enrollment (convalescent samples). To determine duration of viral shedding, real-time, reverse-transcription polymerase chain reaction (rtRT-PCR) test for RSV/A and RSV/B were performed on nasal washes collected at enrollment, day 7 (±1), day 14 (±1), day 21 (±1) and day 28 (±1). At enrollment, an interview was performed to obtain historical information, and medical records were reviewed to extract demographic and clinical data [23,24]. The institutional review boards of the University of Texas MD Anderson Cancer Center and Baylor College of Medicine approved the study protocol and written informed consent was obtained from all the participants.

### 2.2. Biotinylated RSV p27 Peptide 

The consensus sequence of RSV/A p27 peptide ([NH2]ELPRFMNYTLNNTKNTNVTLSKKRKRR[COOH]) was obtained from isolates deposited in GenBank, and sequences from Houston (USA) and Chile [25]. The accuracy of consensus sequence was confirmed by BLAST search at NCBI website by showing 100% query cover and 100% identity to the RSV/A subgroup. The RSV/A p27 peptide was chemically synthesized and biotinylated (Thermo Fisher Scientific). The peptide has a purity of >97% by analytical High Performance Liquid Chromatography. The amino acid content was verified by mass spectrometry. To biotinylate the p27 peptide, an extra Lysine, an aminohexanoic acid (Ahx) spacer, and biotin were sequentially added to the C-terminus of the peptide. The final biotinylated RSV/A p27 peptide sequence was [NH2]ELPRFMNYTLNNTKNTNVTLSKKRKRR-Lys[COOH](Ahx-biotin). 

### 2.3. Biotinylated RSV p27 Monoclonal Antibody

The p27 mouse monoclonal antibody (NVX RSVF7.1) was kindly provided by Dr. Gale Smith (Novavax, MD). The monoclonal antibody (mAb) was biotinylated using Pierce Antibody Biotinylation Kit for immunoprecipitation (Cat. # 90407, Thermo Scientific) with modification to manufacturer instructions. The modification consisted of an initial buffer exchange to purify the monoclonal antibody. The final biotinylated p27 mAb stocks were stored in aliquots at −80 °C. 

### 2.4. Real-Time, Reverse-Transcription Polymerase Chain Reaction (rtRT-PCR)

The RSV/A and RSV/B subtypes in nasal washes were detected by rtRT-PCR. The viral RNA extraction and detection in nasal washes were performed in a CLIA certified Respiratory Virus Diagnostic laboratory (CLIA ID# 45D0919666) as previously described [26].

### 2.5. Enzyme-Linked Immunosorbent Assays (ELISA)

P27 antibodies (serum and mucosal IgG and IgA anti-p27 peptide) were quantified by ELISA. Pierce^TM^ streptavidin coated 96-well plates (Cat. # 15124, Thermo Scientific) were coated with 100 µL of biotinylated p27 peptide at optimized concentration of 100 ng/mL in degassed 1X phosphate-buffered saline (PBS) for 1hr at 36 °C. The plates were washed 4 times with 1X KPL (Cat. # KPL 95059-132, VWR). To generate a standard curve on each plate, 200 µL of p27 mAb at optimized concentration of 10 ng/mL in 2% BSA/degassed 1X PBS were added in duplicate wells followed by 2-fold serial dilutions from 10 to 0.156 ng/mL. Similarly, 200 µL of sera or nasal washes were added in duplicate wells followed by 2-fold serial dilutions from 1:20 to 1:2560 in 2% BSA/degassed 1X PBS. Plates were then incubated at 36 °C for 1 h and washed with 1X KPL. Horseradish peroxidase (HRP)-conjugated goat anti-mouse IgG (Bio-Rad Laboratories, Inc. Richmond, CA) at a dilution of 1:2000 in 2% BSA/degassed 1X PBS was added for p27 mAb standard curve generation. HRP-conjugated goat anti-human IgG (Bio-Rad Laboratories, Inc. Richmond, CA, USA) at a dilution of 1:2000 in 2% BSA/degassed 1X PBS were added for detecting serum and mucosal IgG anti-p27 antibody. HRP-conjugated goat anti-human IgA (Thermo Fisher) at a dilution of 1:4000 in 2% BSA/degassed 1X PBS was added for detecting serum and mucosal IgA anti-p27 antibody. After 1 h incubation at 36 °C, the plates were washed 6 times with 1X KPL and developed with 3,3′,5,5′-Tetramethylbenzidine (TMB) 2-Component Peroxidase Substrate (Kirkegaard and Perry Labs, Gaithersburg, United States) for 18 min in the dark at 25 °C. The reactions were stopped with 0.16 N sulfuric acid. The developed plates were read at 450 nm on a Fluo-Star Optima plate reader within 30 minutes of stopping the reaction. A four-parameter logistic (4PL) regression model was used to calculate the p27 antibody concentrations (ng/mL) based on the dynamic range of the standard curve. The lower limit of detection (LLoD) was 0.2 ng/mL, and a negative sample was assigned a value of 0.1 ng/mL.

### 2.6. P27 Competitive Antibody (P_27_CA) Assay 

Serum and mucosal p27 like antibody (P_27_LA) was measured by a p27 competitive antibody (P_27_CA) assay. One hundred µL of commercial F protein (RSV/A strain A2, Sino Biological) at concentration of 250 ng/mL [23] in 0.05 M carbonate-bicarbonate coating buffer (pH 9.6) was coated onto the Immulon 2HB 96-well plate (Thermo Scientific, Waltham, MA, USA) for 18 h at 4 °C. After three washes with 1× KPL, the plates were blocked for 1 h with 5% milk (Carnation Instant Nonfat Dry Milk) in 1× PBS. A standard curve of p27 mAb (2-fold serial dilutions from 25,000 ng/mL to 24.4 ng/mL) was generated on each plate. Next, 50 µL of 2-fold serial dilutions of test sera or nasal washes (1:10 to 1:1280) in duplicate were added to the coated plates, followed by 50 µL of 100 ng/mL of biotinylated p27 mAb, and 1 hr incubation. After washing, HRP-conjugated streptavidin (SeraCare Life Sciences, Gaithersburg, MD) at 1:2000 dilution in 1X KPL was added for an additional hour incubation. Wells containing biotinylated p27 mAb without samples served as positive controls representing maximum binding of biotinylated p27 mAb to F protein coated on the plates. Wells that were not coated with F protein served as negative controls. TMB color development, stopping the reaction with sulfuric acid, and plate reading were performed the same as described in the p27 ELISA. A 4PL regression model was used to calculate the P_27_LA concentrations (ng/mL) based on the dynamic range of the standard curve by interpolating the concentration of the standards that corresponds to the absorbance value at which the test sample resulted in 50% inhibition. The LLoD was 50 ng/mL for the P_27_CA assay. Samples with concentration below the LLoD were assigned a value of 25 ng/mL. 

### 2.7. Statistical Analysis

Geometric mean concentration (ng/mL) for serum and mucosal antibodies with 95% confidence interval were calculated. A paired t-test was used to determine whether the geometric means of log transformed serum and mucosal antibody concentrations (GMC, log2 ng/mL) differed significantly between acute and convalescent samples. A two-sample t-test was used to determine whether the GMC of log transformed serum and mucosal antibody concentrations differed significantly between RSV/A and RSV/B infected patients, as well as between HCT recipients who shed virus for <14 and ≥14 days. Statistical significance was indicated by *p*-values <0.05. Statistical analyses were performed using the SPSS Statistic 22 (IBM, Armonk, NY, USA).

## 3. Results

### 3.1. Demographic and Clinical Variables of HCT Recipients

Clinical characteristics at enrollment are summarized for all 33 HCT adults in Table 1, stratified by duration of RSV shedding (<14 or >14 days) or by RSV infection subtype (RSV/A or RSV/B). Age, gender, race/ethnicity, absolute neutrophil counts (ANC), absolute lymphocyte counts (ALC), body mass index (BMI), type of transplant, and median time from HCT to RSV infection were comparable between the groups when stratified by duration of RSV shedding or by RSV infection subtype.

### 3.2. Sensitivity and Specificity of P27 ELISA and P27 Competitive Antibody Assay 

**Figure 1:**
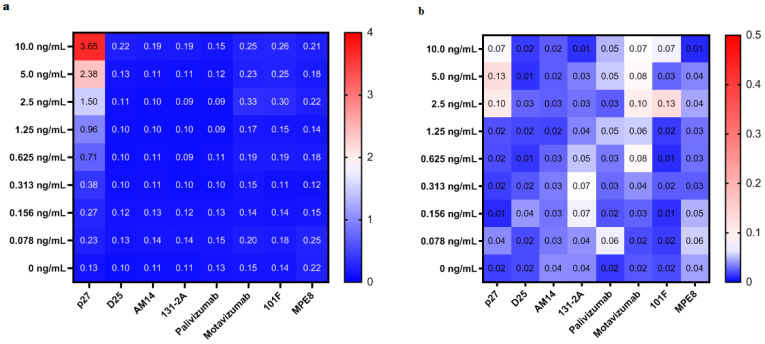
Sensitivity and specificity of ELISA for p27 antibody detection. (**a**) The heat map shows the average optical density (OD) values of p27 monoclonal antibody (p27 mAb) from 3 independent ELISAs when different concentrations of p27 peptide, p27 mAb as well as alternative mAbs were used in the assay. Red and blue indicate strong and weak binding of p27 peptide coated on the plates and the corresponding mAb on the bottom of the figure, respectively. (**b**) The heat map shows the SEs (standard error of the mean) of OD values of p27 mAb from 3 independent ELISAs when different concentrations of p27 peptide, p27 mAb as well as alternative mAbs were used in the assay. Red and blue indicate high and low of SEs calculated, respectively. The heat map was made by GraphPad Prism version 8.3.0 for Windows, GraphPad Software, La Jolla California USA.

The p27 ELISA generated a linear dynamic range with a maximum optical density (OD) value approximately 3.5 and a LLoD of 0.2 ng/mL. MAbs (D25, AM14, MPE8) targeting pre-fusion specific sites (Ø, V and III, respectively), mAb (131-2A) targeting a post-fusion specific site (I), and mAbs (palivizumab, motovizumab, and 101F) binding shared sites (IIa, IIb and IV, respectively) did not cross-react with the p27 peptide (Figure 1a). Figure 1b shows the SEs (standard error of the mean) of OD values of p27 mAb from 3 independent ELISAs when different concentrations of p27 peptide, p27 mAb as well as alternative mAbs were used in the assay. 

**Figure 2:**
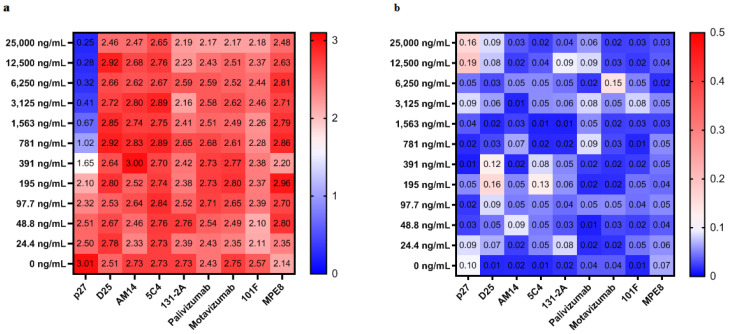
Sensitivity and specificity of p27 competitive antibody (P_27_CA) assay for p27 like antibody (P_27_LA) detection. (**a**) The heat map shows the average OD values of p27 like antibody (P_27_LA) from 3 independent p27 competitive antibody (P_27_CA) assays when different concentrations of p27 mAb and alternative mAbs were used in the assay. Red and blue indicate low and high concentrations of P_27_LA detected, respectively. (**b**) The heat map shows the SEs of OD values of P_27_LA from 3 independent p27 competitive antibody (P_27_CA) assays when different concentrations of p27 mAb and alternative mAbs were used in the assay. Red and blue indicate high and low of SEs calculated, respectively. The heat map was made by GraphPad Prism version 8.3.0 for Windows, GraphPad Software, La Jolla California USA.

Similar specificity was observed with the P_27_CA assay (Figure 2). None of the mAbs to pre-fusion, post-fusion or shared F sites inhibited the binding of biotinylated p27 mAb. A linear dynamic range was also generated with a maximum OD value of 2.5 and a LL_O_D of 50 ng/mL (Figure 2a). Figure 2b shows the SEs of OD values of P_27_LA from 3 independent p27 competitive antibody (P_27_CA) assays when different concentrations of p27 mAb and alternative mAbs were used in the assay.

### 3.3. P27 Antibodies in Sera and Nasal Washes of RSV Infected HCT Recipients 

All 33 RSV infected HCT recipients had detectable IgG anti-p27 antibodies in their acute and convalescent serum samples. There was a 1.9-fold increase in IgG anti-p27 antibodies after the RSV infection, although the convalescent serum concentration was not significantly greater compared to the acute serum concentration (Table 2). Thirty-two of 33 individuals had detectable IgA anti-p27 antibodies in their acute and convalescent serum samples. The serum IgA anti-p27 antibody concentration of the acute and convalescent samples were unchanged. Twenty-eight of 33 RSV infected HCT recipients had detectable levels of serum P_27_LA. An 8.1-fold significant increase of serum P_27_LA was detected in the convalescent serum samples compared to the acute serum samples.

Comparing to serum samples, fewer individuals had detectable IgG or IgA anti-p27 antibodies in their respiratory samples. Twenty of 33 RSV infected HCT recipients had detectable IgG anti-p27 antibodies in their acute and convalescent respiratory samples with a 2-fold decrease in IgG anti-p27 antibodies (Table 2). The convalescent IgG anti p27 antibody concentration, however, was not significantly lower compared to the acute respiratory concentration. Twenty-five of 33 individuals had detectable IgA anti-p27 antibodies in their respiratory samples. Again, a 2-fold decrease in IgA anti-p27 antibodies was detected in the convalescent respiratory concentration, although it was not significantly lower compared to the acute respiratory concentration. Twenty of 33 HCT recipients had detectable levels of P_27_LA in the respiratory samples, and the concentrations were comparable between the acute and convalescent respiratory samples. 

### 3.4. P27 Antibodies in Sera and Nasal Washes of RSV Infected HCT Recipients Who Shed RSV <14 Days and ≥14 Days

We did not observe significant differences in the IgG or IgA anti-p27 antibody or P_27_LA concentration in the acute or convalescent serum samples between RSV infected HCT recipients who shed virus < and ≥14 days (Table 3). The antibody responses, however, appeared differently. For RSV infected HCT recipients who shed virus <14 days, they experienced in the serum a 1.1-, 0.6-, and 9.8-fold increase in IgG anti-p27, IgA anti-p27 and P_27_LA antibodies, respectively, while those who shed virus for ≥14 days had a 2.2-, 1.6- and 6.8-fold rise in IgG anti p27, IgA anti p27 and P_27_LA antibodies, respectively. 

Similar to the results observed in the serum, we did not detect significant differences in the IgG or IgA anti p27 antibody, or P_27_LA concentration in the acute or convalescent respiratory samples between RSV infected HCT recipients who shed virus < and ≥14 days (Table 3). The antibody responses in the respiratory samples appeared comparable between the groups. For RSV infected HCT recipients who shed virus <14 days, they experienced in the respiratory samples a 0.2-, 0.4-, and 1.0-fold increase in IgG anti-p27, IgA anti-p27 and P_27_LA antibodies, respectively, while those who shed virus ≥14 days had 0.9-, 0.5- and 0.8-fold rise in IgG anti-p27, IgA anti-p27 and P_27_LA antibodies, respectively. 

**Figure 3:**
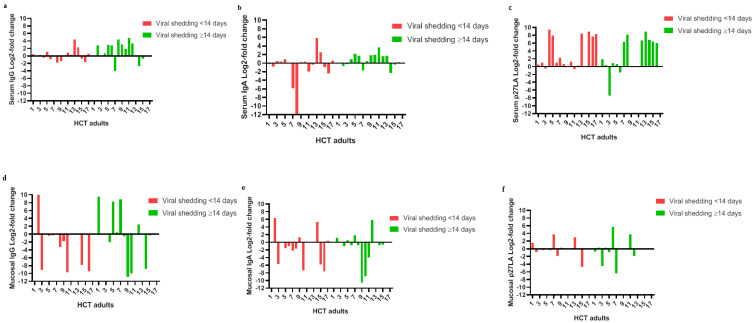
Log2-fold change in p27 antibody concentration between the convalescent to acute samples by RSV infected HCT recipients who shed RSV <14 days (*n* = 17) and >14 days (*n* = 16). For the serum samples, 9 (27.2%), 4 (12.0%) and 14 (42.4 %) HCT recipients (*n* = 33) had a 4-fold rise in the serum IgG, IgA and P_27_LA, respectively (**a**–**c**). For nasal washes, 5 (15.0%), 3 (9.0%) and 4 (12.0%) HCT recipients (*n* = 33) had a >4-fold rise in mucosal IgG, IgA and P_27_LA, respectively (**d**–**f**). In contrast, 9 (27.2%), 8 (24.2 %) and 3 (9.0%) HCT recipients (*n* = 33) had a >4-fold decrease in mucosal IgG, IgA and P_27_LA, respectively (**d**–**f**). In addition, 5 (~30%) HCT recipients who cleared the virus in <14 days (*n* = 17) had a >4-fold decrease in mucosal IgG and IgA anti-p27 antibody concentration, respectively (**d**,**e**). The figure was made by GraphPad Prism version 8.3.0 for Windows, GraphPad Software, La Jolla California USA.

Illustrated in Figure 3 is the log2-fold change in the IgG and IgA anti-p27 antibody concentrations and P_27_LA concentration in the serum and respiratory samples by individuals who shed virus < or ≥14 days. For all of HCT recipients (*n* = 33), 9 (27.2%), 4 (12.0%) and 14 (42.4 %) recipients had a >2 log2-fold (4-fold) rise in the serum IgG anti-p27, IgA anti-p27 and P_27_LA, respectively (Figure 3a–c); 5 (15.1%), 3 (9.0%) and 4 (12.1%) recipients had a >2 log2-fold (4-fold) rise in mucosal IgG anti-p27, IgA anti-p27 and P_27_LA, respectively (Figure 3d–f). Particularly, in either serum or nasal washes from HCT recipients who shed virus <14 days (*n* = 17), we observed an infrequent (~10%) >2 log2-fold (4-fold) rise in IgG (Figure 3a,d) or IgA anti-p27 antibodies (Figure 3b,e).

For all of the HCT recipients (*n* = 33), 2 (6.0%), 4 (12.1%) and 1 (3.0%) recipients had a >2 log2-fold (4-fold) decrease in the serum IgG anti-p27, IgA anti-p27 and P_27_LA, respectively (Figure 3a–c); 9 (27.2%), 8 (24.2 %) and 3 (9.0%) recipients had a >2 log2-fold (4-fold) decrease in mucosal IgG anti-p27, IgA anti-p27, P_27_LA, respectively (Figure 3d–f). This decrease (~30%) in IgG and IgA anti-p27 antibody concentration in respiratory samples was detected mostly in RSV infected recipients who cleared the virus in <14 days (*n* = 17). 

### 3.5. P27 Antibodies in Sera and Nasal Washes of HCT Recipients Infected with RSV/A Versus RSV/B

The RSV/A p27 consensus peptide was used to measure p27 antibodies in HCT recipients infected with RSV/A or RSV/B. Significant differences were not detected in the IgG or IgA anti-p27 antibody concentration or P_27_LA concentration in the acute or convalescent serum samples between HCT recipients who were infected with either RSV/A or RSV/B (Table 4). The antibody responses, however, appeared different. For RSV/A infected HCT recipients, they experienced in the serum a 1.3-, 0.5- and 2.5-fold rise in IgG anti-p27, IgA anti-p27 and P_27_LA antibodies, respectively, while those infected with RSV/B had 1.9-, 1.6-, and 25.0-fold increase in IgG anti-p27, IgA anti-p27 and P_27_LA antibodies, respectively (Table 4).

Again, we did not detect significant differences in the IgG or IgA anti-p27 antibody, or P_27_LA concentration in the acute or convalescent respiratory samples between HCT recipients who were infected with either RSV/A or RSV/B (Table 4). The antibody responses in the respiratory samples appeared comparable between the groups. For RSV/A infected HCT recipients, they experienced in the respiratory samples a 0.4-, 0.7- and 0.9-fold rise in IgG anti-p27, IgA anti-p27 and P_27_LA antibodies, respectively, while those infected with RSV/B had 0.6-, 0.3-, and 0.9-fold increase in IgG anti-p27, IgA anti-p27 and P_27_LA antibodies, respectively. 

## 4. Discussion

RSV F protein is initially synthesized as an inactive monomer containing p27 peptide post-translationally modified with 2 or 3 N-linked glycans [16,27]. To adopt the functional pre-fusion conformation, it is thought that p27 must be cleaved at two furin motifs by a furin-like host protease in the trans-Golgi network as F monomers are transported to the cell plasma membrane [28] where they associate into compact trimers [29]. Therefore, investigators usually assume that RSV p27 is not present on the mature RSV F protein. However, immunization of mice with the F N116Q DNA containing a mutation of the N-glycosylation sequon located within the p27 domain of the RSV F protein elicited enhanced antibody responses and viral protection upon viral challenge [30]. This finding suggests that the removal of N116 glycan unmasked an epitope possibly within the p27 domain that augmented protective antibodies against RSV infection in mice. So far, few studies have investigated the host immune system response to p27 after RSV infections in humans. The present study provided useful data to address the question using RSV infected HCT recipients as the study population. 

We successfully developed ELISA and competitive antibody assays for measuring p27 antibodies and P_27_LA, respectively, in serum and respiratory secretions. The lower limit of detections of p27 ELISA and P_27_CA assays were 0.2 ng/mL and 50 ng/mL, respectively, with no cross-reaction detected with monoclonal antibodies targeting pre-fusion and post-fusion antigenic sites. Using these assays, we detected the IgG and IgA anti-p27 antibodies and P_27_LA in the acute and convalescent serum and nasal wash samples from the majority of RSV infected HCT recipients. It is unclear if p27 is recognized by RSV infected HCT recipients as a free peptide or as a partially cleaved peptide that is still bound to the F protein. The concentration detected was at the nanogram concentration in both serum and nasal wash samples, which was approximately 1000-fold lower than the microgram levels detected to RSV F site-specific domains using competitive antibody assays in the same cohort [23,24]. Although low p27 antibody concentration was detected in our study, our previous study revealed that p27 antibody showed the highest antibody affinity in nasal wash of RSV infected HCT who cleared the virus in less than 14 days [22]. 

In this study, we also observed an infrequent (~10%) four-fold or greater rise in IgG or IgA anti-p27 antibodies in HCT recipients in either serum or nasal washes who shed RSV for <14 days. We had previously demonstrated that the same cohort who cleared virus shedding in the upper respiratory tract within two weeks of enrollment versus those with delayed viral clearance (≥14 days) were able to generate significant rises in neutralizing and F site-specific competitive antibody responses [23,24]. Therefore, the current data suggest that p27 antibody responses in RSV infected HCT recipients did not enhance the neutralizing antibody activity against RSV. Interestingly, approximately 30% of the RSV infected HCT recipients who shed virus for less than 14 days had a four-fold or greater decrease in IgG and IgA anti-p27 antibodies in their respiratory secretions. This suggest that IgG and IgA anti-p27 antibodies are binding to either free viruses or RSV infected cells containing p27, and that p27 antibodies in the respiratory tract is part of the mucosal antibody response in controlling the infection. IgA and IgG anti-RSV antibodies are known to bind RSV infected cells in the upper respiratory tract forming antigen-antibody complexes [31].

Our study has several limitations. The small number of RSV infected adult HCT recipients (*n* = 33) is not representative of adults in the general population, however, it does represent a group that is highly susceptible to the severe consequences of RSV infection. The p27 ELISA and P_27_CA assays used in the current study were against a p27 consensus of RSV/A genotype only, however, RSV/B infected HCT recipients were able to generate a comparable p27 antibody response compared to RSV/A infected HCT recipients. Lastly, the antibody kinetics is limited to a 6-week period. It is possible that a longer observation period will provide greater clarity on the kinetics of the p27 antibody response. 

## 5. Conclusions

In summary, we developed a p27 ELISA and P_27_CA assay for quantifying the serum and mucosal IgG and IgA, and P_27_LA against the RSV/A p27 peptide. The two assays had lower limit of detection of 0.2 ng/mL and 50 ng/mL, respectively with no cross-reaction detected with monoclonal antibodies targeting pre-fusion and post-fusion antigenic sites. Using these assays, we discovered that p27 antibodies were detectable at nanogram concentrations in sera and nasal washes in RSV infected HCT recipients. The p27 antibodies in serum did not appear to enhance neutralizing antibody activity. However, approximately a third of the RSV infected HCT recipients had a 4-fold or greater decrease in their mucosal IgG and IgA anti-p27 antibodies suggesting antibodies are binding to either free viruses or RSV infected cells containing p27, and that p27 antibodies in the respiratory tract were part of the mucosal antibody response in controlling the infection. In conclusion, we provided novel knowledge regarding p27 antibody responses in HCT recipients, and will need further investigation to understand the significant role of p27 antibody responses in other age groups. 

## Figures and Tables

**Table 1:** Demographic and clinical characteristics of respiratory syncytial virus (RSV) infected hematopoietic cell transplant (HCT) recipients, stratified by duration of RSV shedding (< 14 vs. ≥14 days) and by RSV type (RSV/A vs. RSV/B).

Variable	Duration of RSV Shedding		*p*-Values ^a^	RSV Type		*p*-Values ^a^
	<14 Days (*n* = 17)	≥14 Days (n=16)		RSV/A (*n* = 16)	RSV/B (*n* =17)	
Age (y), mean ± SD	52.4 ± 14.6	50.3 ± 18.2	0.72	52.5 ± 15.4	50.4 ± 17.4	0.71
Female, % (n)	62.5 (10)	37.5 (6)	0.30	56.3 (9)	43.8 (7)	0.49
Race, % (n)			0.38			0.54
White	41.2 (7)	58.8 (10)		47.1 (8)	52.9 (9)	
Black	75.0 (3)	25.0 (1)		25.0 (1)	75.0 (3)	
Hispanic	50.0 (5)	50.0 (5)		50.0 (5)	25.0 (5)	
Asian	100.0 (2)	0.0 (0)		100.0 (2)	0.0 (0)	
ANC ^b^ at enrollment, mean ± SD (n)	3.35 ± 2.32 (16)	2.82 ± 1.92 (15)	0.50	3.35 ± 2.52	2.82 ± 1.62 (15)	0.49
ALC ^c^ at enrollment, mean ± SD (n)	1.29 ± 0.69 (16)	1.10 ± 1.09 (15)	0.57	1.25 ± 0.78	1.16 ± 1.03 (15)	0.79
BMI ^d^, mean ± SD	27.3 ± 6.0	28.3 ± 7.5	0.70	27.8 ± 8.3	27.8 ± 5.0	>0.99
Type of transplant, % (n)			0.12			0.12
autologous	77.8 (7)	22.2 (2)		22.2 (2)	77.8 (7)	
allogeneic	41.7 (10)	58.3 (14)		58.3 (14)	41.7 (10)	
Time from HCT (d), median (range)	251 (6–945)	99.5 (5–1067)	0.23	272.5 (6–1067)	157 (5–486)	0.20

^a^ Chi-squared test or Fisher’s exact test or two-sample t-test or Wilcoxon rank-sum test; ^b^ ANC = absolute neutrophil count × 1000 (per cm^3^ of blood); ^c^ ALC = absolute lymphocyte count × 1000 (per cm^3^ of blood); ^d^ BMI = body mass index (kg/m^2^).

**Table 2:** p27 Antibody concentrations in acute and convalescent sera and nasal washes from RSV infected HCT adults.

p27 Antibody Test	Acute (*n* = 33)	Convalescent (*n* = 33)	Fold Change	*p* Value ^b^
**Serum IgG ELISA**	413.1 (234.3, 719.1) ^a^	653.2 (387.6, 1069.5)	1.9	0.075
**Serum IgA ELISA**	36.6 (18.2, 66.1)	34.0 (13.8, 69.4)	0.9	0.84
**Serum P_27_CA**	288.3 (109.5, 752.6)	2361.5 (1043.7, 4910.5)	8.1	<0.001
**Mucosal IgG ELISA**	2.7 (0.8, 9.6)	1.3 (0.4, 3.7)	0.5	0.463
**Mucosal IgA ELISA**	2.4 (1.0, 5.6)	1.1 (0.5, 2.3)	0.5	0.280
**Mucosal P_27_CA**	146.3 (75.0, 287.4)	134.6 (77.0, 251.6)	0.9	0.762

^a^ Geometric mean concentration (ng/mL) for serum and mucosal antibodies (95% Confidence Interval) in RSV infected HCT adults. ^b^ Paired t-test for difference in means of antibody concentration (log2 ng/mL) between acute and convalescent sera from RSV infected HCT adults.

**Table 3:** p27 antibody concentrations in RSV infected HCT adults who shed RSV <14 days and ≥14 days.

p27 Antibody Test	Serum	<14 Days (*n* = 17)	Fold Change	≥14 Days (*n* = 16)	Fold Change	*p*-Value ^b^
**Serum IgG ELISA**	Acute	405.0 (174.3, 946.4)^a^	1.1	422.0 (203.6, 946.0)	2.2	0.942
	Convalescent	461.6 (24.7, 922.0)		944.7 (439.5, 1920.2)		0.183
**Serum IgA ELISA**	Acute	43.8 (14.6, 105.8)	0.6	30.3 (11.1, 60.1)	1.6	0.587
	Convalescent	24.4 (6.1, 74.1)		48.2 (15.9, 108.0)		0.413
**Serum P_27_CA**	Acute	484.5 (118.6, 1833.0)	9.8	166.1 (52.1, 639.4)	6.8	0.271
	Convalescent	4756.7 (1593.3, 10,822.9)		1122.2 (318.5, 3027.5)		0.071
**Mucosal IgG ELISA**	Acute	2.9 (0.4, 21.1)	0.2	2.6 (0.4, 13.6)	0.9	0.935
	Convalescent	0.7 (0.1, 4.1)		2.3 (0.4, 12.3)		0.574
**Mucosal IgA ELISA**	Acute	1.7 (0.5, 6.0)	0.4	3.5 (0.9, 11.2)	0.5	0.430
	Convalescent	0.7 (0.2, 2.5)		1.6 (0.4, 5.2)		0.772
**Mucosal P_27_CA**	Acute	197.5 (71.0, 563.1)	1.0	106.4 (46.0, 283.0)	0.8	0.378
	Convalescent	200.1 (84.7, 470.9)		88.3 (41.2, 213.7)		0.195

^a^ Geometric mean concentration (ng/mL) for serum and mucosal antibodies (95% Confidence Interval) in RSV infected HCT adults. ^b^ Two-sample t test for differences in means of antibody concentration (log2 ng/mL) in HCT adults who shed RSV <14 days and ≥14 days.

**Table 4:** P27 antibody concentrations between RSV/A versus RSV/B infected HCT adults.

P27 Antibody Test	Serum Type	RSV/A (*n* = 16)	Fold Change	RSV/B (*n* = 17)	Fold Change	*p* Value ^b^
**Serum IgG ELISA**	Acute	462.7 (187.8, 1176.0) ^a^	1.3	371.3 (189.8, 746.1)	1.9	0.698
	Convalescent	593.0 (262.9, 1206.1)		715.4 (340.6, 1549.5)		0.730
**Serum IgA ELISA**	Acute	50.3 (18.1, 119.6)	0.5	27.2 (10.0, 61.1)	1.6	0.363
	Convalescent	26.8 (7.6, 73.7)		42.5 (11.0, 117.7)		0.580
**Serum P_27_CA**	Acute	1081.8 (250.1, 3565.9)	2.5	83.0 (33.42, 267.2)	25.0	0.006
	Convalescent	2710.9 (898.7, 6992.4)		2073.9 (501.5, 6835.9)		0.744
**Mucosal IgG ELISA**	Acute	4.9 (1.7, 32.8)	0.4	1.6 (0.3, 8.1)	0.6	0.378
	Convalescent	1.8 (0.3, 10.1)		1.0 (0.2, 4.9)		0.854
**Mucosal IgA ELISA**	Acute	1.5 (0.4, 5.9)	0.7	3.7 (1.3, 10.7)	0.3	0.339
	Convalescent	1.0 (0.3, 3.4)		1.2 (0.4, 3.6)		0.567
**Mucosal P_27_CA**	Acute	173.0 (73.5, 450.1)	0.9	125.0 (47.1, 351.7)	0.9	0.645
	Convalescent	166.1 (67.0, 482.8)		110.4 (53.2, 233.8)		0.522

^a^ Geometric mean conc., (ng/mL) for humoral and mucosal antibodies (95% Confidence Interval) in RSV infected HCT adults. ^b^ Two-sample t-test for difference in means of antibody concentration (log2 ng/mL) between RSV/A and RSV/B infected HCT adults.

## Meeting Participation

The information of the manuscript was presented in 5th ReSViNET Conference RSVVW 2019 in Accra, Ghana.
